# Serum IgG levels in children 6 months after SARS-CoV-2 infection and comparison with adults

**DOI:** 10.1007/s00431-021-04124-w

**Published:** 2021-05-22

**Authors:** Silvia Bloise, Alessia Marcellino, Alessia Testa, Anna Dilillo, Saverio Mallardo, Sara Isoldi, Vanessa Martucci, Maria Teresa Sanseviero, Emanuela Del Giudice, Donatella Iorfida, Flavia Ventriglia, Riccardo Lubrano

**Affiliations:** Dipartimento Materno Infantile, UOC di Pediatria e Neonatologia Ospedale Santa Maria Goretti, Polo Pontino, Sapienza Università di Roma, Latina, Italy

**Keywords:** Antibody response, Children, COVID-19, Immunity

## Abstract

Since the outbreak of SARS-CoV-2 among the population has occurred quite recently, there is a lack of evidence on the long-term duration of antibody response, especially in children. It is therefore crucial to clarify this aspect, considering its implications in the development of successful surveillance strategies, therapies, and vaccinations. The aim of this study was to assess the antibody response in a children group after SARS-CoV-2 infection, and to compare it with that of their parents affected by SARS-CoV-2 infection. We enrolled 12 children and their parents, both groups being affected by COVID-19 in April 2020. In the children’s group, we collected real-time RT-PCR cycle threshold (Ct) values and gene characterization of first nasal-throat swab at the time of diagnosis (T0); 30 days after the diagnosis (T30), we performed blood tests to detect anti-SARS-CoV-2 IgM and IgG. Finally, 180 days after the diagnosis (T180), we measured anti-SARS-CoV-2 IgG in both children and parents. In children, antibody levels declined significantly at 180 days (T180) after first measurement (T30). There were no significant differences in IgG level related to age, sex, and clinical manifestations. We found a significant correlation between IgG titers at T30 and Ct value of gene N. Children showed a lower level of antibodies against SARS-CoV-2 at T180 compared to their parents.

*Conclusion*: Antibody responses in children waned 180 days after SARS-CoV-2 infection, and at the same time, their parents showed a different antibody response to the virus. These results highlight that serological tests should be used with caution in surveillance strategies among the general population.

**Table:** 

**What is known:** • *Currently is not known how long antibody response will be maintained or if it protects from reinfection*. • *Recent reports in adults suggest that antibodies to SARS-CoV-2 declined several months after infection, but data are missing in pediatric age*.	
**What is new:** • *We showed that antibody responses to SARS-CoV-2 wane several months after infection also in children with quantitative differences in antibody levels between children and adults*. • *In this context, serological tests should be used with caution in surveillance strategies*.

**What is known:**

• *Currently is not known how long antibody response will be maintained or if it protects from reinfection*.

• *Recent reports in adults suggest that antibodies to SARS-CoV-2 declined several months after infection, but data are missing in pediatric age*.

**What is new:**

• *We showed that antibody responses to SARS-CoV-2 wane several months after infection also in children with quantitative differences in antibody levels between children and adults*.

• *In this context, serological tests should be used with caution in surveillance strategies*.

## Background

At the end of 2019, a novel coronavirus was identified as responsible for a cluster pneumonia in Wuhan, Hubei Province, China. The evaluation with real-time polymerase chain reaction (RT-PCR) of bronchoalveolar lavage samples from a patient led to identify the etiologic cause: Severe acute respiratory syndrome coronavirus 2 (SARS-CoV-2), a single-stranded RNA virus, belonging to the b-coronavirus genus. In February 2020, the World Health Organization named the disease associated to its infection as the 2019 novel coronavirus disease (COVID-19), and in March, the outbreak was officially declared as a public health emergency of international concern [1].

Most of the published reports have primarily focused on the disease features; however, more recently, the attention and research shifted to the investigation of the host immune response to the virus.

Similar to other viral infections, SARS-CoV-2 stimulates an innate immune response and a subsequent adaptive immune response with development of neutralizing antiviral T cell and antibody [2].

Neutralizing antibodies are crucial for the establishment of a protective immunity [3, 4], but is not yet currently known how long antibody responses will be maintained or if humoral immune response protects from reinfection. In fact, the dynamics of humoral immune responses in pediatric age still remain unknown.

There is a growing need to clarify these issues, considering the lowest susceptibility to COVID-19 of children compared with adult population [5–13]; therefore, study their immune response could be important to understand the mechanism capable of controlling the infection, and to provide further information for the development of successful surveillance strategies, therapies and vaccines.

For these reasons, we conducted a study to evaluate the antibody response in a children group after SARS-CoV-2 infection, also comparing the antibody response with that of their parents, also affected by SARS-CoV-2 infection.

## Materials and methods

This was a prospective cohort study conducted at the Pediatric Unit of Santa Maria Goretti Hospital, in Latina—Sapienza University of Rome (Polo Pontino) between May 2020 and October 2020.

Recruitment was conducted within the geographical area of the province of Latina by general pediatricians, who were engaged via email invitations to participate in the study. All children who resulted positive for SARS-CoV-2 infection at nasopharyngeal swab test in April 2020 were offered to participate in the study if at least one of their parents was infected as well; also, in parents the infection was detected by nasal-throat swab test for SARS-CoV-2 nucleic acid by Real-time reverse transcription PCR (rRT-PCR). All those who consented to the study protocol, which included serology testing in children 1 month after the initial infection, and a serology testing both in children and their parents 6 months after the initial infection, were consecutively enrolled.

All participants were interviewed on symptoms and signs of infection and on the possible existence of other family members infected with SARS-CoV-2. We investigated the presence of comorbidities; risk factors, as obesity (defined by BMI ≥30 kg/m^2^) and smoking (smoker was defined by having smoked at least 100 cigarettes in his or her lifetime, being a smoker at the time of the interview, or having quit smoking for less than 6 months).

Furthermore, we investigated the use of medications influencing antibody response.

In the children’s group, we collected real-time-PCR cycle threshold (Ct) values and gene characterization of first nasal-throat swab at the time of diagnosis (T0); 30 days after the diagnosis (T30), we performed another nasal-throat swab and blood tests to detect anti-SARS-CoV-2 IgM and IgG. Finally, 180 days after the diagnosis (T180), we measured anti-SARS-CoV-2 IgM and IgG both in children and parents.

The primary aim of our study was to describe the dynamic changes of serum antibody levels against SARS-CoV-2 30 days and 180 days after the infection in children.

Secondary aims were to evaluate possible demographic, clinical, and laboratory factors influencing the antibody response in our pediatric cohort and to compare it with that of their parents, 180 days after the infection.

### Laboratory test

The infection was detected by nasal-throat swab test for SARS-CoV-2 nucleic acid by real-time reverse transcription PCR (rRT-PCR) targeting three genes: envelope protein (E), RNA-dependent RNA polymerase (RdRp), and nucleocapsid protein (N).

The STARMag 96 × 4 Universal Cartridge Kit (Seegene Inc.) was used to extract total RNA, and gene fragments were detected by Allplex TM 2019 n-CoV assay (Seegene Inc.).

The cycle threshold (Ct) values of rRT-PCR represent the number of replication cycles required to produce a fluorescent signal, with lower Ct values representing higher viral RNA loads.

According to the manufacturer’s instructions, samples with a Ct value < 40 were regarded as SARS-CoV-2 detected, and a Ct value < 40 for only one of the three targets was considered positive.

The serum antibodies against SARS-CoV-2 were detected by chemiluminescent immunoassay (CLIA), using the iFlash Immunoassay Analyzer (YHLO Biotech Co., Ltd, Shenzhen, China).

The iFlash-SARS-CoV-2 IgM and IgG assay used present a sensitivity of 97.3% and specificity 96.3% [14].

These antibodies are against nucleocapsid and spike protein.

The antibody levels were expressed as arbitrary unit per ml (AU/ml). The results ≥10 AU/ml were considered positive, while the results < 10 AU/ml negative.

### Statistical analysis

The statistical analysis was performed with JMP® 15.2.0 program for Mac (SAS Institute Inc.).

For all variables the approximation of population distribution to normality was tested by Kolmogorov-Smirnov One-Sample Test and statistics for kurtosis and symmetry. Because the results were asymmetrically distributed all data were expressed as median, 25° and 75° quartiles and consequently presented as box and whisker plot. For the analysis of the differences between the group of the study the non-parametric Wilcoxon tests was used. For the correlation of continuous variables Pearson’s or Spearman’s coefficient was calculated according to the distribution.

A descriptive analysis using percentage values was performed for qualitative variables.

A *p* value < 0.05 was considered significant.

## Results

### Demographic, clinical, and virological characteristics of the patients

We enrolled 12 children and 12 parents. In the children group, 7 subjects were male and 5 were females; the median age was 13.37 (9.6–14.3) years.

In the children group two patients were asymptomatic (17%), while 10 (83%) suffered from a mild clinical condition, showing fever (80%), acute upper respiratory symptoms (25%), gastrointestinal symptoms (25%), and other symptoms, as myalgia, ageusia, anosmia and headache (60%).

Children comorbidities were allergic rhinitis (one patient) and coeliac disease (one patient).

In the parent group, 7 subjects were males and 5 were females; the median age was 47 (40.5–51.2) years.

All parents were symptomatic, showing fever (100%), upper respiratory symptoms (83%), gastrointestinal symptoms (25 %), and other symptoms, as myalgia, ageusia, anosmia, and headache (83%)*.* Three parents showed cough and dyspnea and were hospitalized with diagnosis of pneumonia.

Parent’s comorbidities were allergic rhinitis (one parent) and hypertension (two parents).

Furthermore, four parents were smokers.

No patients enrolled were taking medications influencing antibody response

Patients’ demographic and clinical characteristics are summarized in Table 1.

**Table 1:** Patients’ demographic and clinical characteristics

	Children	Parents
Gender (males/females)	7/5	7/5
Age (median (25–75°)) Children (years) Parents (years)	13.37 9.6–14.3	47 40.5–51.2
Caucasians (*N* (%))	12 (100%)	12 (100%)
Comorbidities (*N* (%))	2 (17%) Allergic rhinitis, coeliac disease	3 (25%) Allergic rhinitis, hypertension
Risk factors (*N* (%)) - Obesity (BMI≥30) - Smoking	0 (0%) 0 (0%)	0 (0%) 4 (33%)
Symptoms - Fever (TC ≥ 37.5 C°) (*N* (%)) - Upper respiratory symptoms (*N* (%)) - Gastrointestinal symptoms (*N* (%)) - Others (myalgia, ageusia, anosmia, and headache) (*N* (%)) - Cough and dyspnea (*N* (%))	10 (80%) 3 (25%) 3 (25%) 7 (60%) 0 (0%)	12 (100%) 10 (83%) 3 (25%) 10 (83%) 3 (25%)
Hospitalized (*N* (%))	0 (0%)	3 (25%)

The genomic characterization and RT-PCR cycle threshold (Ct) values of nasal-throat swabs of children at T0 are summarized in Table 2.

**Table 2:** The genomic characterization and RT-PCR cycle threshold (Ct) values of nasal-throat swabs of children at T0

Children	E gene-Ct	Gene RdRp-Ct	Gene N-Ct
1	37.05	40	40
2	21.64	21.88	24.38
3	40	40	36.63
4	40	40	38.70
5	40	40	35.88
6	40	33.85	35.55
7	17.24	19.34	20.07
8	40	33.40	34.22
9	25.86	26.93	29.27
10	30.44	31.45	33.50
11	30.54	34.78	33.77
12	40	18.17	40

### Levels of serum antibodies at T30 and at T180 in children

**Fig. 1:**
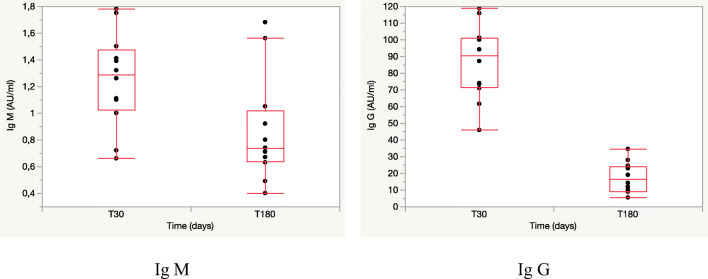
Decline of IgM and IgG antibody levels in children 180 days after SARS-CoV-2 infection. The boxes include value of median, 25° and 75° quartiles; the whiskers include 10° and 90° quartiles

As shown in Fig. 1, the IgM and the IgG levels declined significantly (IgM: *p*< 0.0262; IgG: *p*< 0.0001) at 180 days (T180) compared to previous measurement (T30).

At T30, the median IgM level was 1.29 (1.02–1.47) AU/ml; at T180, it was 0.74 (0.64–1.01) AU/ml.

At T30, the median IgG level was 90.61 (71.5–101) AU/ml; at T180, it was 16.53 (9.1–24.1) AU/ml

At T30, no child had detectable IgM levels, while all children (100%) showed positive IgG levels.

At T180, no child had detectable IgM levels, while 9 children (75%) showed positive IgG levels.

### Relationship between antibody response and demographic, clinical, and virological characteristics in children

**Fig. 2:**
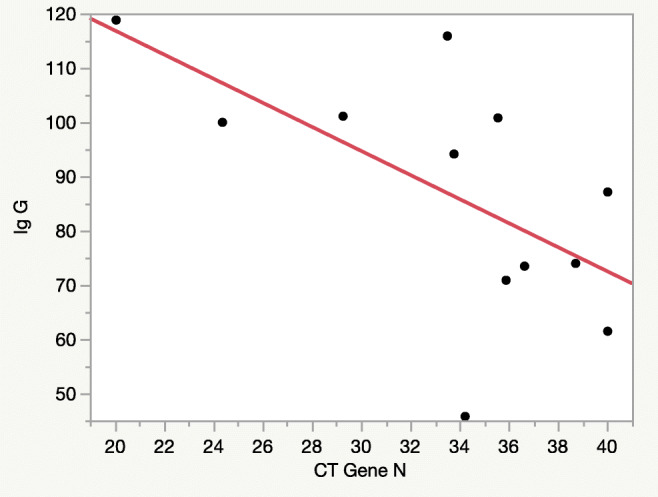
Correlation between anti-SARS-CoV-2 IgG titers at T30 and Ct of gene N in children

There were no significant difference in IgG levels related to age (*r*^2^ =0.27, *p*=0.07), sex (*p*=0.56), presence of symptoms (fever, *p*=0.87; respiratory symptoms, *p*=0.40; gastrointestinal symptoms, *p*=0.87; others symptoms, *p*=0.63) and duration of symptoms (*r*^2^ =0.10, *p*=0.63). Instead, analyzing the correlation between IgG level and virological characteristics, we observed that children positive for E gene have significantly higher titers of IgG levels (100.51 (116.59–92.36) AU/ml vs 72.16 (57.54–80.66) AU/ml, *p* < 0.02)), while the IgM levels did not show significant variations (1.29 (1.01–1.42) AU/ml vs 1.26 (0.91–1.76) AU/ml, *p*=0.77)). Moreover, we found a significant correlation between IgG titers at T30 and Ct value of gene N (*r*^2^ = 0.38, *p*<0.033) (Fig. 2), while correlations between IgG titers at T30 and Ct value of gene E and gene RdP were not significant (*r*^2^ 0.026, *p*=0.61; *r*^2^ 0.026, *p*=0.095).

### Comparison of children’s antibody levels, IgG, and IgM with their parents at T180

**Fig. 3:**
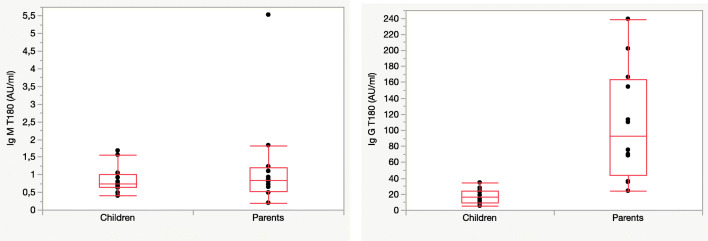
Comparison of children’s IgM and IgG antibody levels with their parents at T180. The boxes include value of median, 25° and 75° quartiles; the whiskers include 10° and 90° quartiles

At T180, children showed lower level of IgG antibodies against SARS-CoV-2 compared to their parents (*p* <0.0001), while IgM levels were similar (*p*=0.93) (Fig. 3).

In children, the median IgM level was 0.74 (0.64–1.01) AU/ml; in parents, the median IgM level was 0.83 (0.53–1.19) AU/ml.

In children, the median IgG level was 16.5 (9.1–24.1) AU/ml; in parents, the median IgG level was 92.7 (44.1–163.3) AU/ml.

At T180, no child or parent had detectable IgM levels, while all parents (100%) and 9 children (75%) showed positive IgG levels.

## Discussion

There is a lack of evidence regarding the long-term duration of antibody response against SARS-CoV-2, especially in children.

In adult patients, recent reports suggest that antibody response to SARS-CoV-2 declined significantly in the 3 months following SARS-CoV-2 infection [15–19].

In study, we observed a significant decay of antibody response also in pediatric age 6 months after SARS-CoV-2 infection. Nevertheless, we think that these data should not be considered alarming, since the absence of specific antibodies does not mean absence of immune memory. In fact, our immune system has several strategies to ensure an immune memory, as T cell.

Currently, different studies showed that T cells are also implied during SARS-CoV-2 infection and polyfunctional T cells (PFC) with a stem-like memory phenotype were detectable both in convalescent patients and in blood samples from people who had not been exposed to the virus [20–22].

Differently from adults, whose antibody levels seem to correlate with the severity of the disease [23–25], in our pediatric cohort we found no correlation in antibody levels related to presence of symptoms; This may be in line with what has been reported in the literature, considering that all our symptomatic children suffered from a mild clinical condition and nobody developed severe complications of disease.

Instead, analyzing the virological characteristics of our patients, we observed that children positive for E gene had significantly higher titers of IgG level at T30.

We hypothesized that the highest levels of antibodies in this group of children were due to a higher viral load. In fact, the majority of children with E gene was also positive for the other two genes researched; furthermore, we found a significant correlation with the Ct of N gene. This aspect sustained our hypothesis, considering the relation between Ct value and viral copy number value [26, 27].

Finally, our results showed quantitative difference in the anti-SARS-CoV-2 specific antibody response between children compared with their parents, supporting the results of a recent study of Weisberg et al. [28], where the authors demonstrated a reduced protective serological response in children compared to adults. These data support the hypothesis of a distinct immune response in pediatric age that could be at the base of lower susceptibility to SARS-CoV-2 in children. This could be related to a more robust innate immune response resulting in an efficacious viral clearance and consequent reduced acquired immune response [29]. Since the outbreak occurred many authors sustained this hypothesis [30, 31] and recently the research in this area has allowed to identify biological and cellular characteristics at the base of age-related different host immune responses. Perce et al. [32] demonstrated that pediatric patients had higher serum concentrations of interleukin-17A and interferon-γ shortly after infection, contributing to immune protection, particularly against lung disease. Instead, other authors focused on early and more effective polyclonal innate or IgM memory B cell response [33], or increased number of naïve T cells [28] that make children capable of a more rapid reaction to new pathogens.

Our study presents several limitations: the small sample size that was composed by children with mild infection, not requiring hospitalization; the lack of discrimination between antibodies against N and S protein of SARS-CoV-2; the lack of the data related to antibodies level of adults at 30 days after the infection.

However, given that parents and their children are often infected in the same household, we arguably recorded the immune response against the same strain of virus.

## Conclusion

Our study showed that antibody responses wane after SARS-CoV-2 infection also in pediatric age.

However, pending new evidences about the duration of immunity, we think that preventive measures, as universal facial masking, should be implemented also in children to limit the spread of infection [34].

Furthermore, the reduced antibody response in children compared with their parents confirmed a distinct infection course between the two age groups and corroborated the hypothesis of an age-related immune response, with a more robust innate immune response in the pediatric population.

Finally, analogously to adults, the rapid decline of antibody levels in children should prompt to cautiously consider serological tests for surveillance strategies among the general population.

## Data Availability

All data and materials support published claims and complied with field standards

## Abbreviations

COVID-19: Coronavirus disease 2019
E: Envelope protein
RT-PCR: Real-time reverse transcription polymerase chain reaction
Ct: Real-time-PCR cycle threshold
SARS-CoV-2: Severe acute respiratory syndrome coronavirus 2

## Code availability

N/A
